# Supplementary material for: Nitrifying niche in estuaries is expanded by the plastisphere
Source: Nat Commun. 2024 Jul 12;15:5866. doi: 10.1038/s41467-024-50200-8 (PMC11245476; doi:10.1038/s41467-024-50200-8)
Supplement: Supplementary file 1 — Supplementary Information [file 41467_2024_50200_MOESM1_ESM.pdf]

Supporting Information to

Nitrifying niche in estuaries is expanded by plastisphere

Xiaoxuan Su[1,2#], Xinrong Huang[1,3#], Yiyue Zhang[1#], Leyang Yang[1,3], Teng Wen[4], Xiaoru Yang[1], Guibing Zhu[3,5], Jinbo Zhang[4,6], Yijia Tang[7], Zhaolei Li[2], Jing Ding[8], Ruilong Li[9], Junliang Pan[10], Xinping Chen[2], Fuyi Huang[1], Matthias C. Rillig[11,12], Yong-guan Zhu[1,3,5] *

[1] Key Laboratory of Urban Environment and Health, Ningbo Observation and Research Station, Institute of Urban Environment, Chinese Academy of Sciences, 1799 Jimei Road, Xiamen 361021, China

[2] Interdisciplinary Research Center for Agriculture Green Development in Yangtze River Basin, Southwest University, Chongqing, 400715, China

[3] University of the Chinese Academy of Sciences, 19A Yuquan Road, Beijing 100049, China

[4] School of Geography, Nanjing Normal University, Nanjing 210023, China

[5] Research Center for Eco-Environmental Sciences, Chinese Academy of Sciences, Beijing 100085, China

[6] Liebig Centre for Agroecology and Climate Impact Research, Justus Liebig University, Germany

[7] School of Life and Environmental Sciences, The University of Sydney, NSW 2015, Australia

[8] School of Environmental and Material Engineering, Yantai University, Yantai 264005, P. R. China

[9] School of Marine Science, Guangxi University, Nanning 530004, China

[10] School of Electrical Engineering, Chongqing University, Chongqing 400044, China

[11] Freie Universität Berlin, Institute of Biology, Berlin, Germany

[12] Berlin-Brandenburg Institute of Advanced Biodiversity Research, Berlin, Germany

# These authors contributed equally to this work.

*Corresponding author: **Yong-guan Zhu** (ygzhu@rcees.ac.cn); Phone: 86-592-6190997; fax: +86 592-6190977; Address: 1799 Jimei Road, Xiamen 361021, China.

Supplementary methods and codes

1. Gas Chromatography settings

In this study, N_2O and O_2 were analyzed using an Agilent 7890 Gas Chromatograph (GC) equipped with a ^{63}Ni electron capture detector (ECD) and a thermal capture detector (TCD). The sensitivity of the GC is calculated by the coefficient of variation (CV) and is considered to be acceptable when CV <10%. O_2 and N_2O is determined after separation on a Porapak Q column (1.8 m, 80/100 mesh) at 80 °C. N_2O is analyzed with the ECD (350 °C) using argon/methane (90/10, P10) as a carrier gas. O_2 is analyzed with TCD using nitrogen carrier gas. The carrier flow rate was set at ~27 cm^3 min^{-1}, which yielded a retention time of ~2.5 min and ~4.6 min for appearance of N_2O and O_2 peaks, respectively.

2. Procedures for DNA extraction from biofilms

To compare the biomass and microbial communities across different biofilms, we standardized the sample preparation by processing 3 g of each biofilm material, including plastic debris, wood debris, stone debris, and glass balls, after incubations. The DNA was extracted using a FastDNA™ Kit (MP, Biomedicals, USA) according to the manufacturer instructions. The detailed procedures are as follows:

(a) We prepared 3 g of each material known to form biofilms.

(b) These materials were transferred into 150-mL sterile serum bottles filled with 30 mL of sterile water.

(c) The bottles were shaken at 15°C and 200 rpm for 6 hours.

(d) Biofilms were detached from the materials using a knife as completely as possible, resulting in a 30-mL biofilm suspension.

(e) From this suspension, 1.2 mL was taken and added to the sample tubes of

the MP kit, and DNA extraction proceeded according to the kit guidelines.

3. Codes for Monte Carlo sampling

```
%---Calculations of Mean values and errors of N2O-SP for two endmembers---
Amean=mean(ab(:,1)); %A: SPND
Astd=std(ab(:,1));
Bmean=mean(ab:,2)); %B: SPNN
Bstd=std(ab(:,2));

%------Main Program-------
num_sample=40000; %Total stimulations
num_sample1=10000;%effective stimulations
result=zeros(12,4);
for nnn=1:12
num_data=nnn;
j=1;
f12=ones(num_sample1,2);
%------Random sampling of SP-------
A=normrnd(Amean,Astd,[1 num_sample]);
B=normrnd(Bmean,Bstd,[1 num_sample]);

%------Calculations of fND, fND------
for i=1:num_sample
H=[1 A(i) B(i)];
if max(data(num_data,:)*inv(H))<1 && min(data(num_data,:)*inv(H))>-1
```

```
f12(j,:)=data(num_data,:)*inv(H);
j=j+1;
end
if j>num_sample1
    break
end
end

%------Results output-------
f12mean=mean(f12);
result(nnn,1:2)=mean(f12);
result(nnn,3)=sum(sum((f12-f12mean).^2))/(num_sample1-1);
end

%------Figuring-------
% hold on
% for i=1:10000
```

Table S1. Ranges and average values of SP-N2O, which were used for calculation.

Endmembers	N_2O-SP range (‰)	Average N_2O-SP (‰)	References
ND	-13.6~1.9	-5.9	(1, 2)
NN	32~38.7	35	(2, 3)

Note: ND: nitrifier denitrification; NN: NH_2OH oxidation.

Table S2. Primers for targeting microbial communities and nitrifiers in the plastisphere and surrounding seawater.

Genes	Primers	Primers (5'-3')	PCR conditions
AOA *amoA*[4]	archaea-*amo*AF	STAATGGTCTGGCTTAGACG	5 min at 95°C, 35 cycles consisting of 45 s at 94°C, 1 min at 53°C and 1 min at 72°C.
	archaea-*amo*AR	GCGGCCATCCATCTGTATGT	
AOB *amoA*[4]	*amo*A-1F	GGGGTTTCTACTGGTGGT	5 min at 95°C, 35 cycles consisting of 30 s at 94°C, 30 s at 55°C and 1 min at 72°C.
	*amo*A-2R	CCCCTCKGSAAAGCCTTCTTC	
NOB *Nitrospina* 16S (qPCR)[5]	130F	GGGTGAGTAACACGTGAATAA	94°C for 3min; 35×(94°C for 15s; 57.5°C for 15s; 72°C for 30s; 77°C for 7s)
	282R	TCAGGCCGGCTAAMCA	
NOB *Nitrospira* 16S (qPCR)[6]	616F	AGAGTTTGATYMTGGCTC	95°C for 4min; 35 cycles×(94°C for 30s; 56°C for 30s; 72°C for 60s; 72°C for 7min)
	1158R	CCCGTTMTCCTGGGCAGT	
NOB *Nitrospira nxrB* (qPCR)[7]	169F	TACATGTGGTGGAACA	95°C for 5min; 35×(95°C for 40s; 56°C for 40s; 72°C for 90s; 72°C for 10min)
	638R	CGGTTCTGGTCRATCA	
NOB Nitrobacter *nxrA* (qPCR)[8]	F1370 F1	CAGACCGACGTGTGCGAAAG	3 min at 94°C followed by 35 cycles of 94°C for 30s, annealing at 55°C for 45s and at 72°C for 45s with terminal elongation at 72°C for 5min
	F2843 R2	TCCACAAGGAACGGAAGGTC	
Comammox *amo*A clade A[9]	comaA-244F	TAYAAYTGGGTSAAYTA	50 °C for 2 min and 95 °C for 10 min, followed by 40 cycles of 15 s at 95 °C, 30 s at 52 °C, and 40 s at 72 °C
	comaA-659R	ARATCATSGTGCTRTG	
Comammox *amo*A clade B[9]	comaB-244F	TAYTTCTGGACRTTYTA	
	comaB-659R	ARATCCARACDGTGTG	
16S rRNA[10]	515F	GTGCCAGCMGCCGCGG	95°C for 3 min, 30 cycles of 95°C for 30s, 55°C for 30s and 72°C for 45s, 72°C for 10min
	907R	CCGTCAATTCMTTTRAGTTT	
16S rRNA[11]	Arch344F	ACGGGGYGCAGCAGGCGCGA	95°C for 3 min, 30 cycles of 95°C for 30s, 55°C for 30s and 72°C for 45s, 72°C for 10min
	Arch915R	GTGCTCCCCCGCCAATTCCT	

Words in grey indicate the amplification failure.

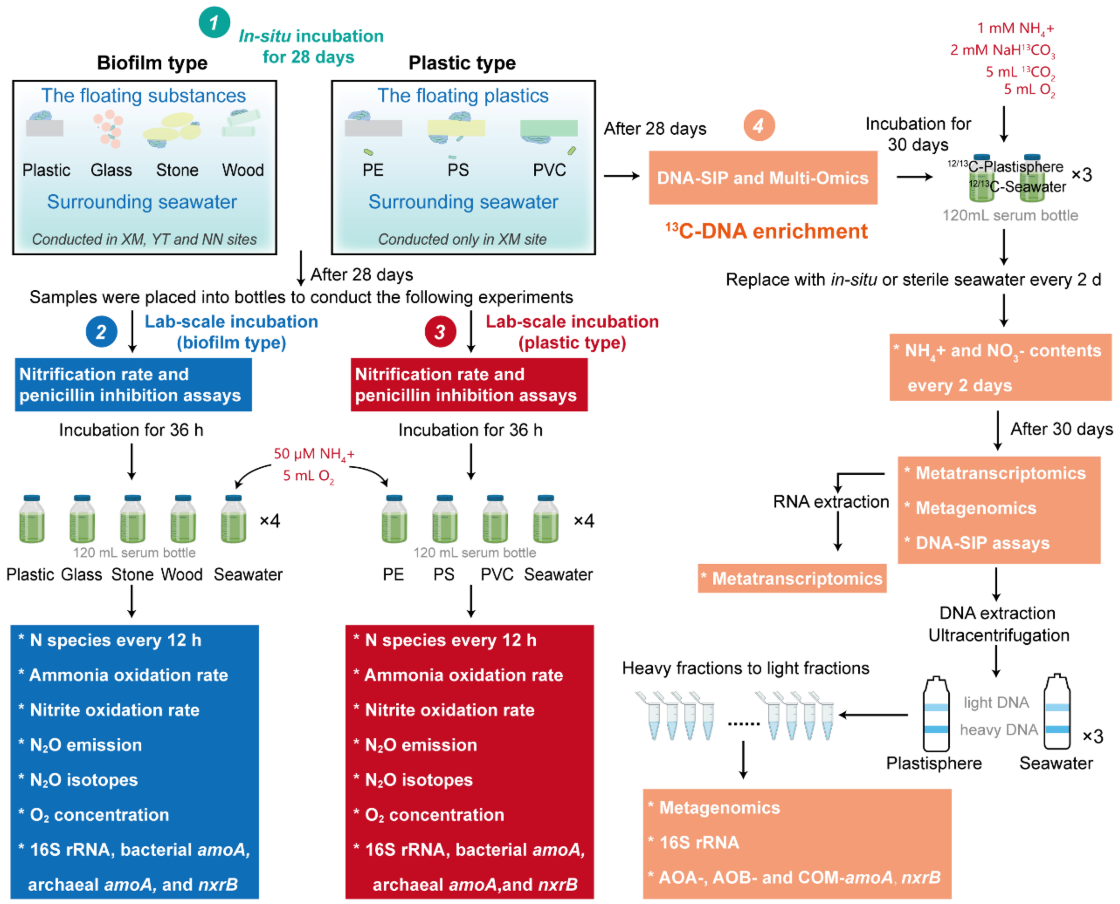

Fig. S1. Experimental workflow of this study. The experiments in this study includes **1** *in-situ* (28 days, Experiment 1), **2** biofilm type-based lab-scale incubations (36 hours, Experiment 2), **3** plastic type-based lab-scale incubations (36 hours, Experiment 3), and **4** ^{13}C-substrate flush feeding incubation of DNA-SIP assays (Experiment 4).

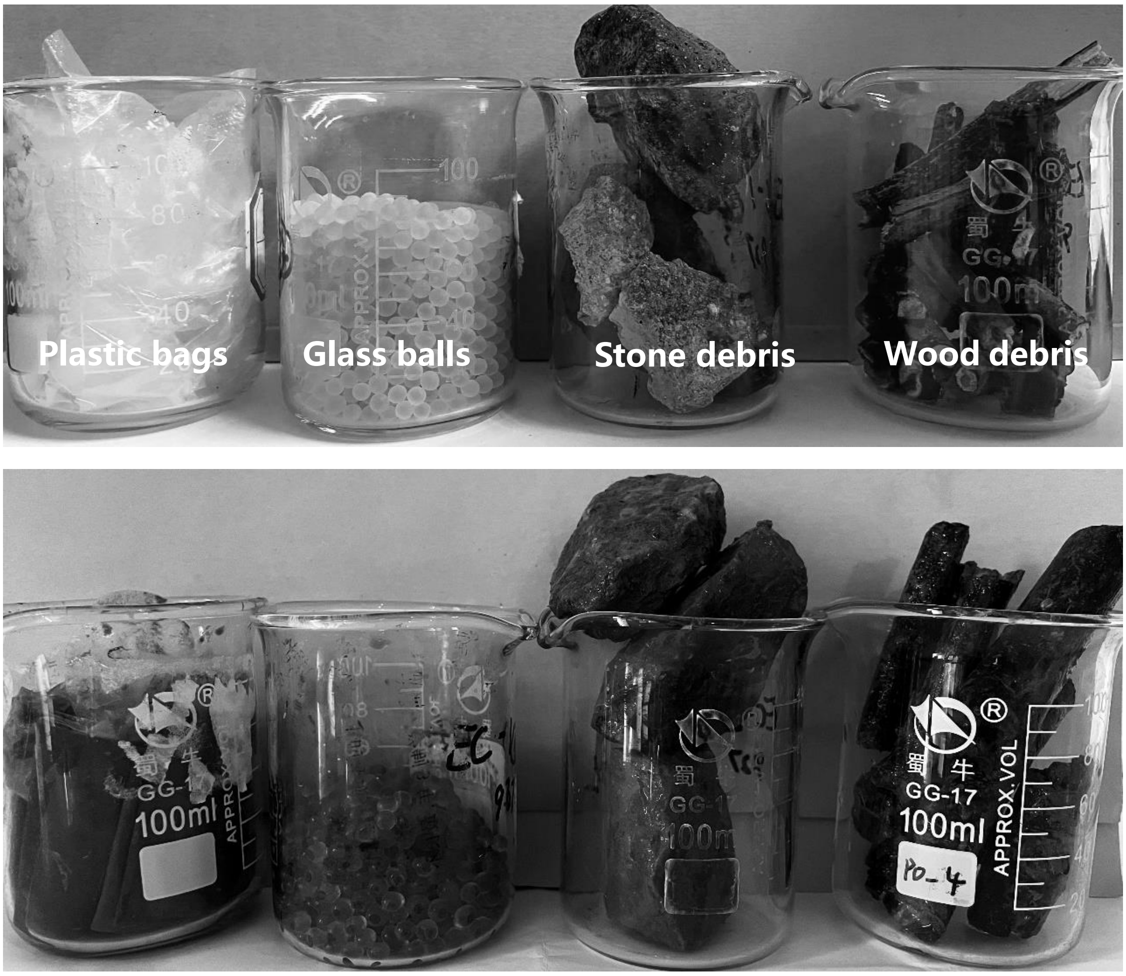

Fig. S2. Photo images of biofilms on the surfaces of plastic bags, glass balls, stone debris and wood before and after in-situ incubations. Microbial aggregates found to be attached to the stone surfaces were denser than on the plastic surfaces. The biofilms were less on the glass surfaces, and were loose and easily dispersed on the wood surfaces. This figure was taken when the biofilms were harvested.

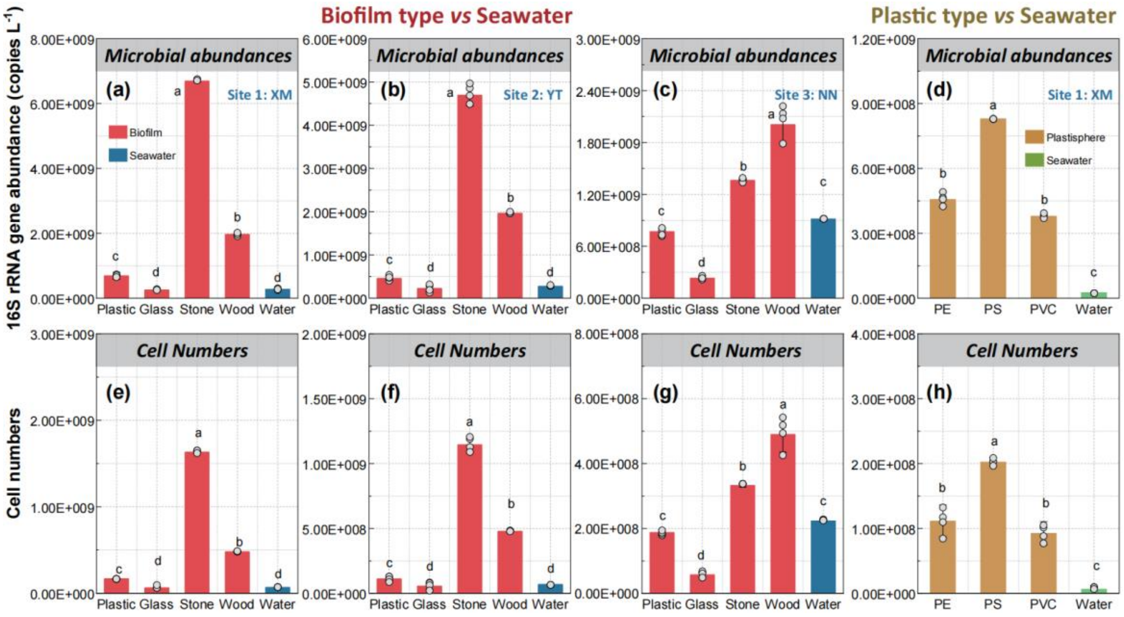

Fig. S3. Microbial abundances and cell numbers after the 36-h incubation in Experiment 2 (biofilm type-based) and Experiment 3 (plastic type-based). (a)-(d)16S rRNA-based microbial abundances (n=4, biological replicates) at XM, YT and NN sites; **(e)-(h)** cell numbers (n=4, biological replicates) at XM, YT and NN sites. Microbial cell numbers were calculated by 16S rRNA abundance / 4.1 on the basis of Ribosomal RNA Operon Copy Number Database. Data are presented as mean value ± standard deviation. Different letters indicate the significant differences (one-way ANOVA, P<0.001-0.039).

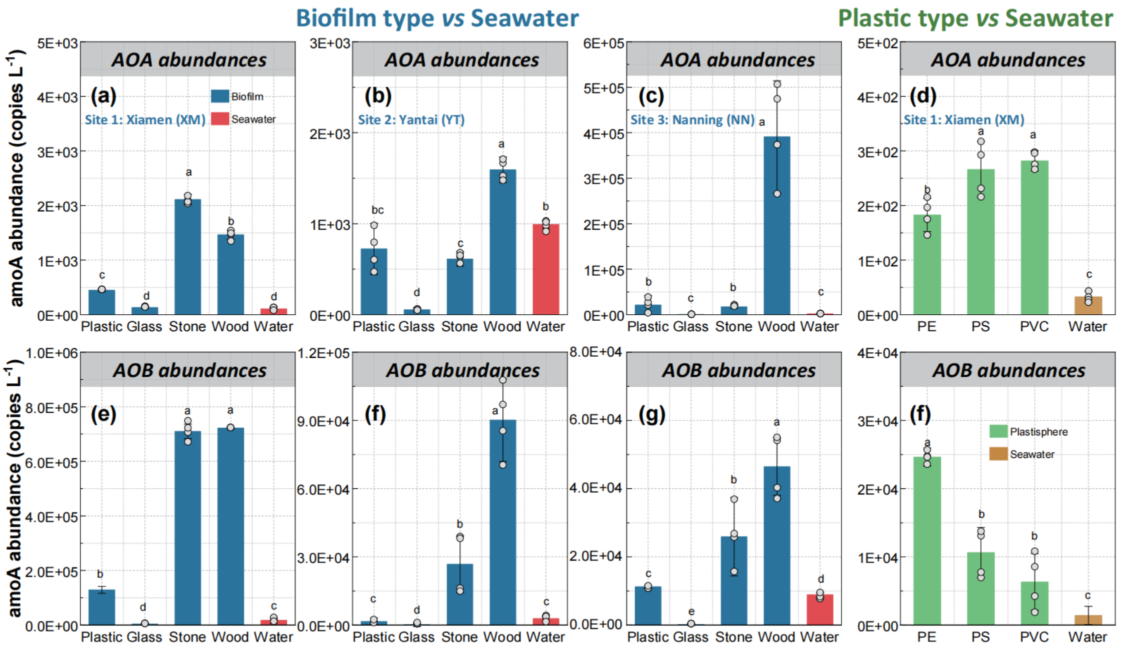

Fig. S4. Abundances of nitrifiers including AOA and AOB after the *in-situ* incubation. (a)-(d) AOA abundances (n=4, biological replicates) at XM, YT and NN sites; **(e)-(h)** AOB abundances (n=4, biological replicates) at XM, YT and NN sites. No COM nitrifiers were amplified. Data are presented as mean value ± standard deviation. Different letters indicate the significant differences (one-way ANOVA, P<0.001-0.047).

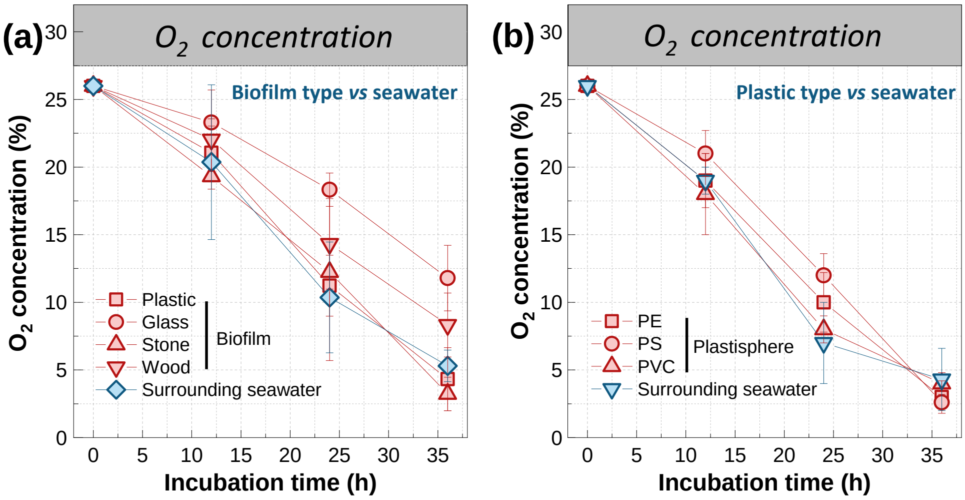

Fig. S5. Changes of O_2 concentrations during the 36-h incubation. (a) The Experiment 2 (biofilm type-based), including plastic, glass, stone, wood and surrounding seawater. **(b)** The Experiment 3 (plastic type-based), including PE, PS, PVC plastics and surrounding seawater. The initial concentration of O_2 was 26%. Data are presented as mean value ± standard deviation (n=4, biological replicates).

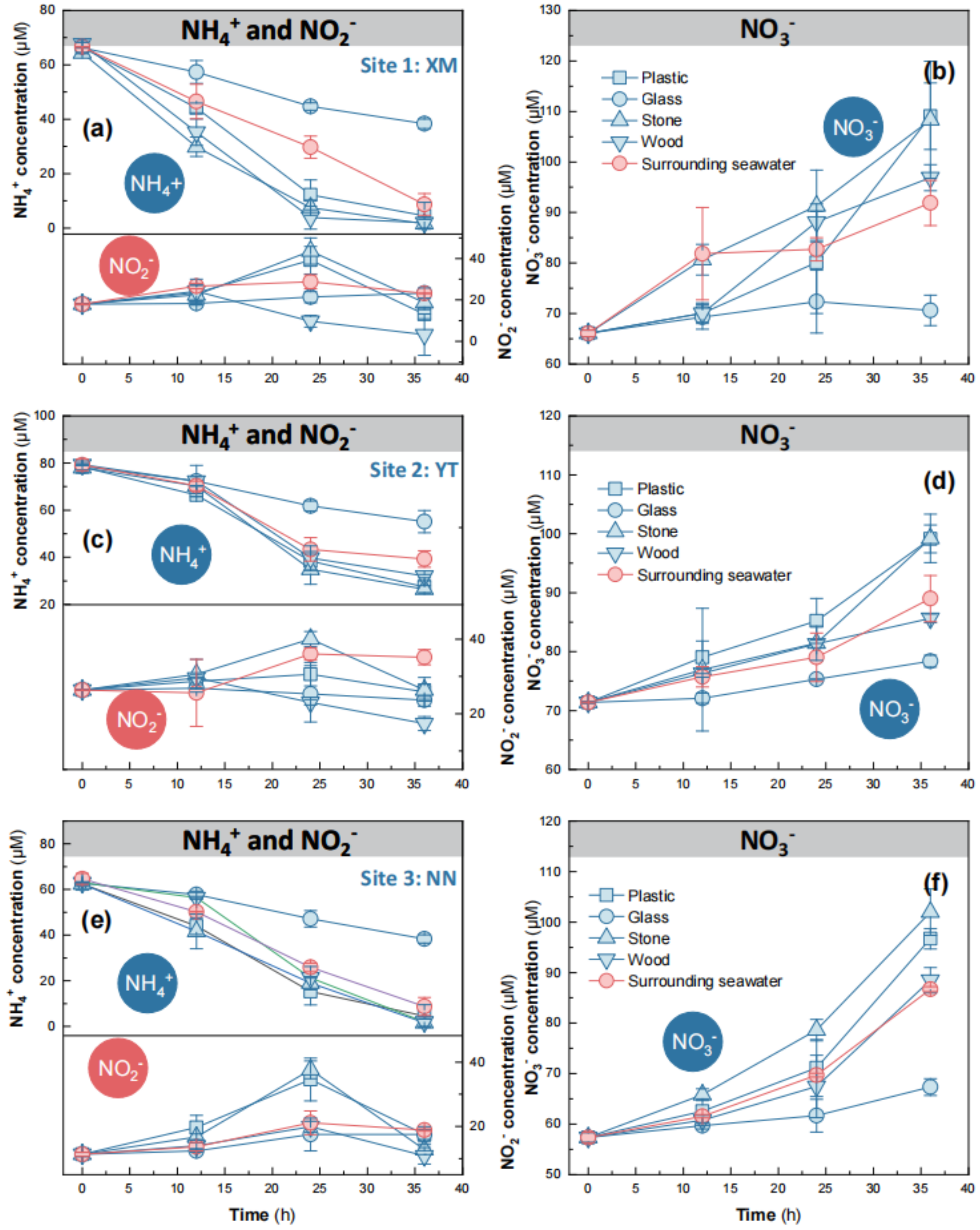

Fig. S6. Changes of NH_4^+, NO_2^- and NO_3^- concentrations during the 36-h incubation (Experiment 2) in the biofilm groups and the surrounding seawater group. (a), **(c)** and **(e)** are the variations of NH_4^+ and NO_2^- concentrations at XM, YT and NN sites, respectively. **(b)**, **(d**) and **(f)** are the variations of NO_3^- at XM, YT and NN sites, respectively. From the perspective of nitrification stoichiometry, the decrease in NH_4^+ and increase in NO_3^- appeared to be unbalanced. This is likely due to microbial assimilation and biofilm adsorption of NH_4^+. Data are presented as mean value ± standard deviation (n=3, biological replicates).

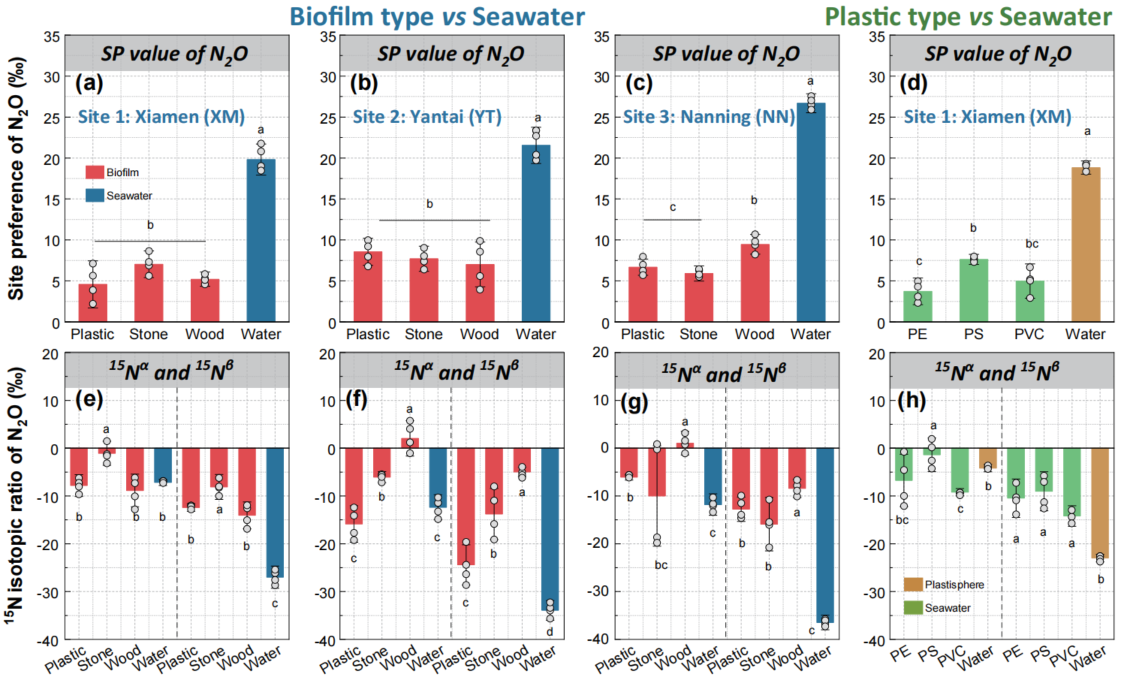

Fig. S7. Isotopic natural abundances of site preference (SP)-N_2O, $\delta^{15}N^{\alpha}$-N_2O, $\delta^{15}N^{\beta}$-N_2O. (a) and **(e)** are based the biofilm-type incubations at XM site. **(b)** and **(f)** are based the biofilm-type incubations at YT site. **(c)** and **(g)** are based the biofilm-type incubations at NN site. **(d)** and **(h)** are based the plastic-type incubations at XM site. Data are presented as mean value ± standard deviation. Different letters indicate the significant differences (n=4, biological replicates, one-way ANOVA, P<0.001-0.041).

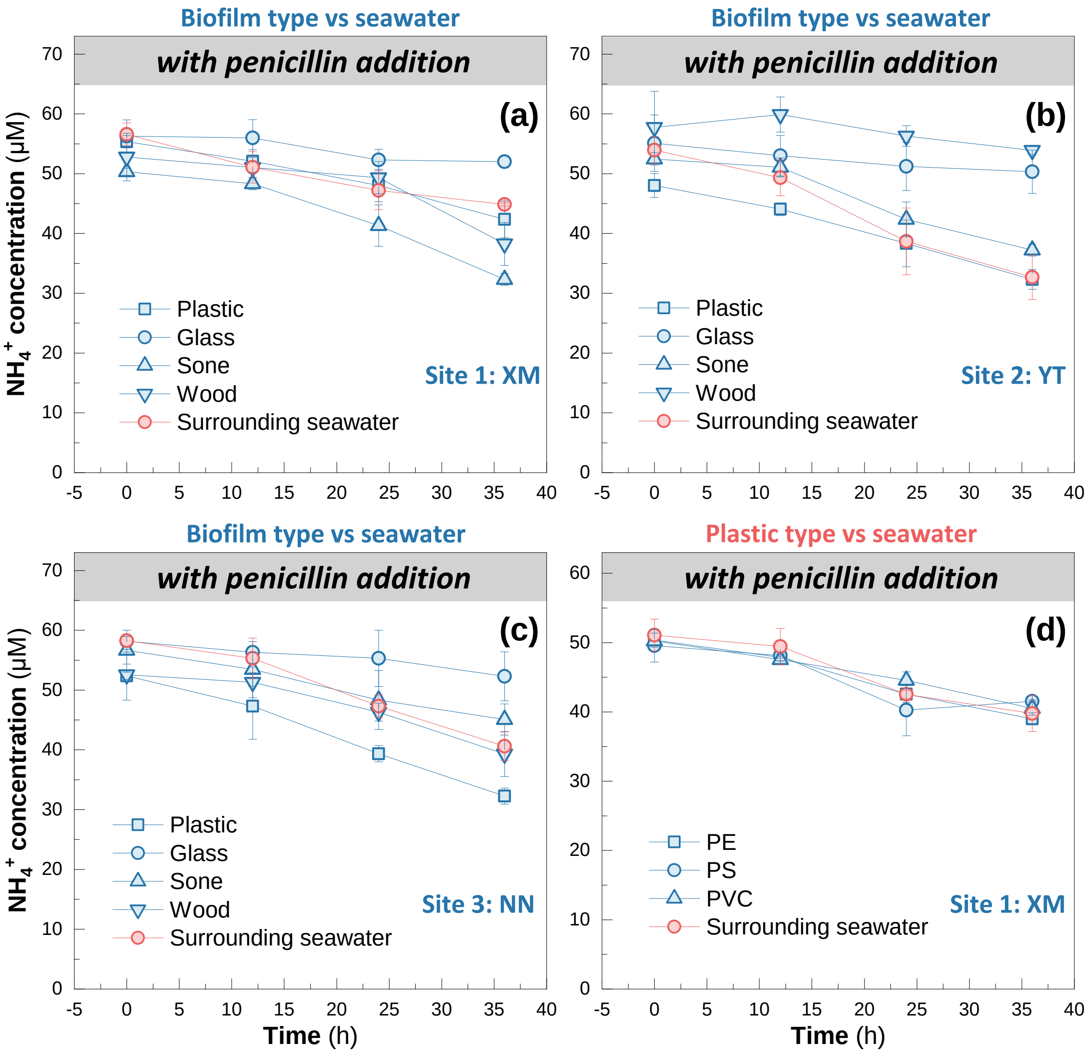

Fig. S8. Changes of NH_4^+ concentration during the 36-h incubation in Experiment 2 (biofilm type-based) and Experiment 3 (plastic type-based). **(a)** XM siter; **(b)** YT site; (**c**) NN site; (**d**) XM site. Data are presented as mean value ± standard deviation (n=3, biological replicates).

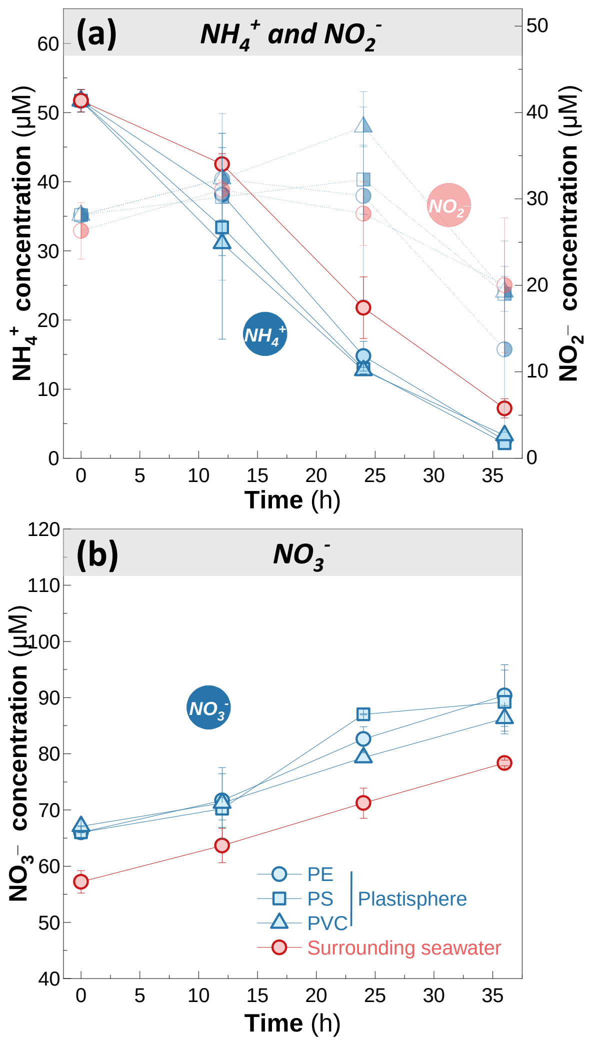

Fig. S9. Changes of NH_4^+, NO_2^- (a) and NO_3^- (b) concentrations during the 36-h incubation (Experiment 3) in the plastisphere groups and the surrounding seawater group. Data are presented as mean value ± standard deviation (n=3, biological replicates).

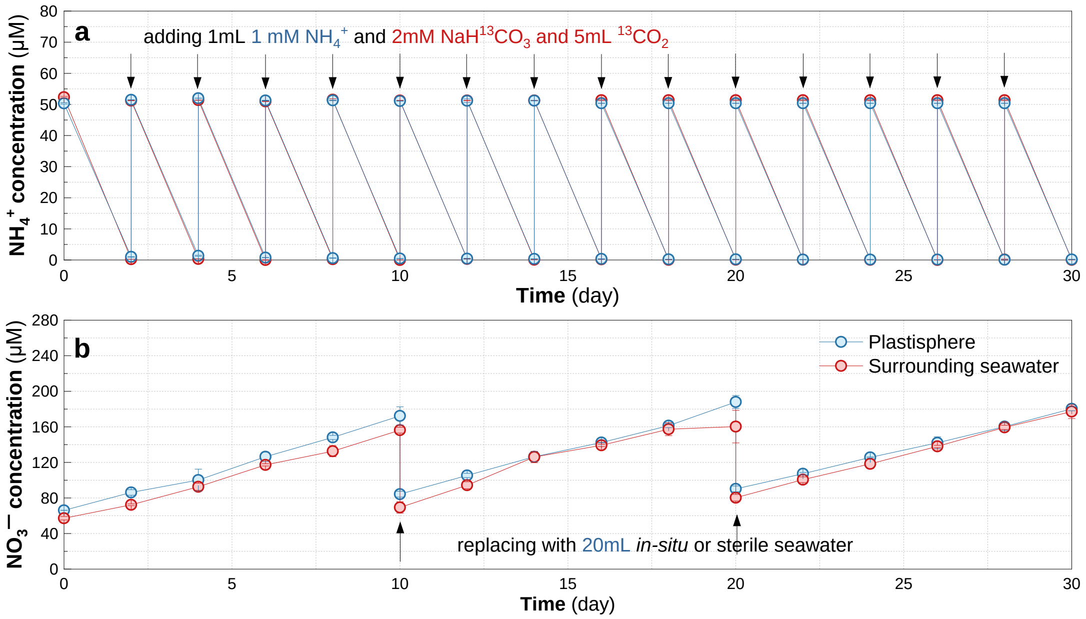

Fig. S10. Experiment 4: Flush-feeding incubations with $^{13/12}CO_2$ and $NaH^{13/12}CO_3$ for DNA-SIP. (a) NH_4^+ concentration; **(b)** NO_3^- concentration. We resupplied with $(NH_4)_2SO_4$, $NaHCO_3$, O_2 and CO_2 every 2 days. NH_4^+, NO_2^-, NO_3^- concentrations were measured before each resupplement. To avoid the accumulation of NO_3^-, we replaced with fresh sterile or *in-situ* seawater every 10 days. We only showed the results of ^{13}C-lablled microcosms due to the similar trends in ^{12}C-lablled microcosms.

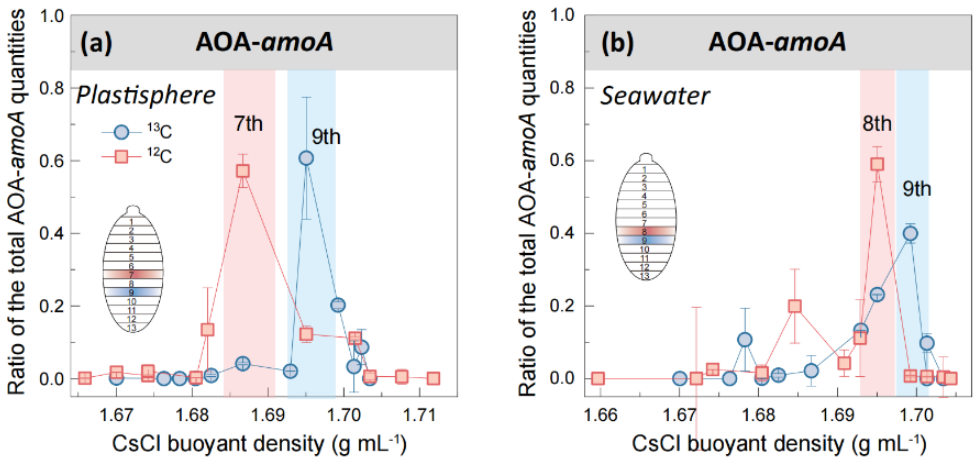

Fig. S11. DNA-SIP. The ^{13}C-DNA and the ^{12}C-DNA of AOA are shown by qPCR of AOA-*amoA* across the CsCl buoyant density gradient in the DNA-SIP assays. The results are normalized using the ratio of AOA-*amoA* copy number in each DNA fraction to the total AOA-*amoA* copy numbers of all fractions in each sample. **(a)** plastisphere; **(b)** seawater. Data are presented as mean value ± standard deviation (n=3, biological replicates).

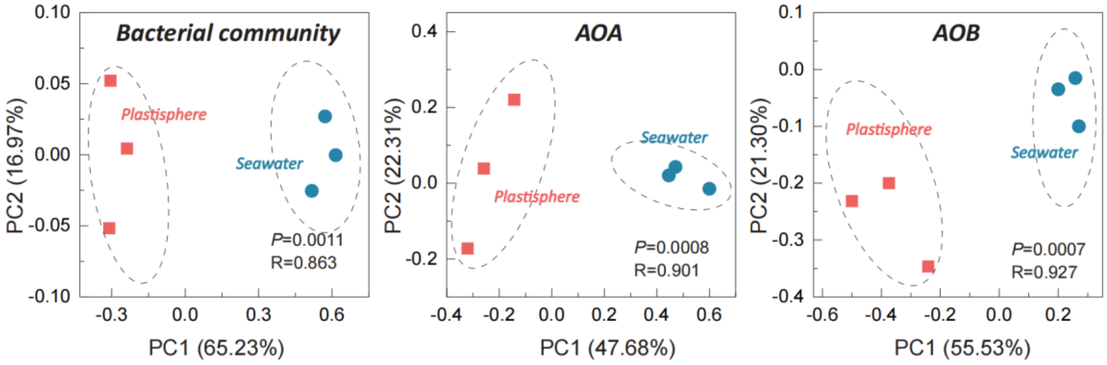

Fig. S12. β-diversity of bacterial community (16S rRNA), AOA community (archaeal-*amoA*), and AOB community (bacterial-*amoA*). Principal coordinate analysis (PCoA) along with permutational multivariate analysis of variance (PERMANOVA) based on Bray-Curtis distances were conducted. As COM community was only detected in the plastisphere, we did not present the data in this figure.

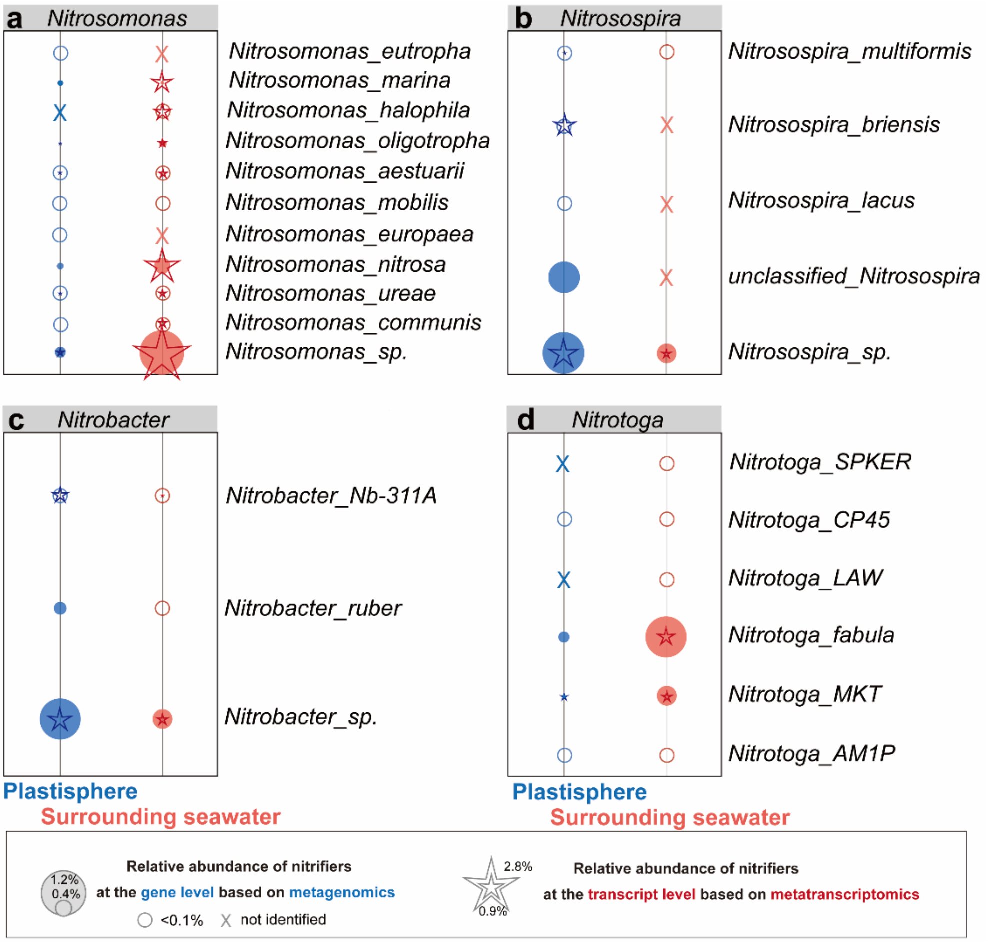

Fig. S13. The relative abundances and expressions of active nitrifier species based on metagenomics and the metatranscriptomics in the plastisphere and the surrounding seawater. (a) *Nitrosomonas*; **(b)** *Nitrosospira*; **(c)** *Nitrobacter*, and **(d)** *Nitrotoga*. Notably, the abundances of *Nitrobacter_Nb-311A* and *Nitrosospira_briensis* in the plastisphere were lower than 0.1% at the gene level, but they had a high transcriptional activity (0.51% and 0.68%).

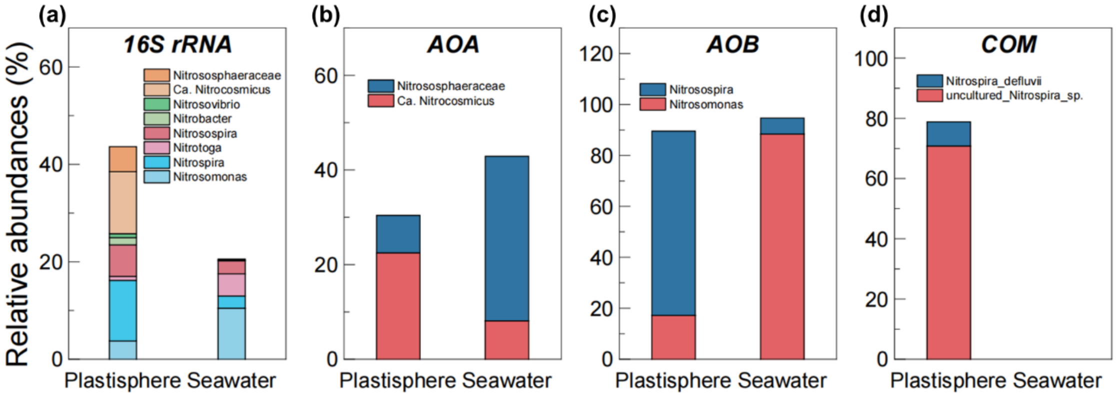

Fig. S14. The relative abundances of active nitrifiers based on amplicon sequencing in the plastisphere and the surrounding seawater. (a) 16S rRNA based nitrifier compositions; **(b)** Archaeal-*amoA* (AOA) containing nitrifiers; **(c)** Bacterial-*amoA* (AOB) containing nitrifiers; (**d**) Comammox-*amoA* (COM) containing nitrifiers.

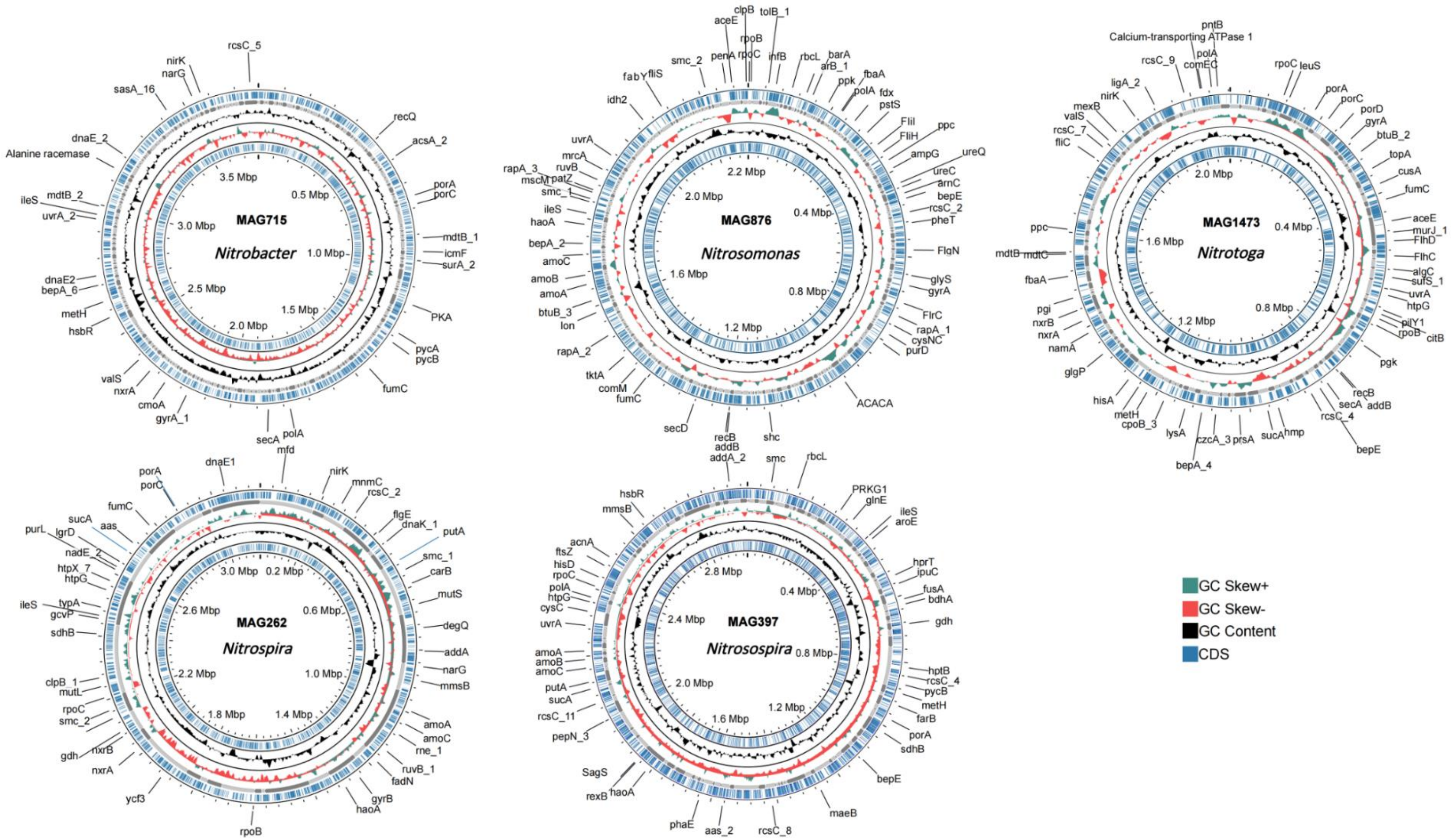

Fig. S15. The circular genomes of the five nitrifying MAGs. MAG262 (*Nitrospira*), MAG397 (*Nitrosospira*), MAG715 (*Nitrobacter*), MAG876 (*Nitrosomonas*), MAG1473 (*Nitrotoga*). The genomes with GC content, GC skew and CDS are shown.

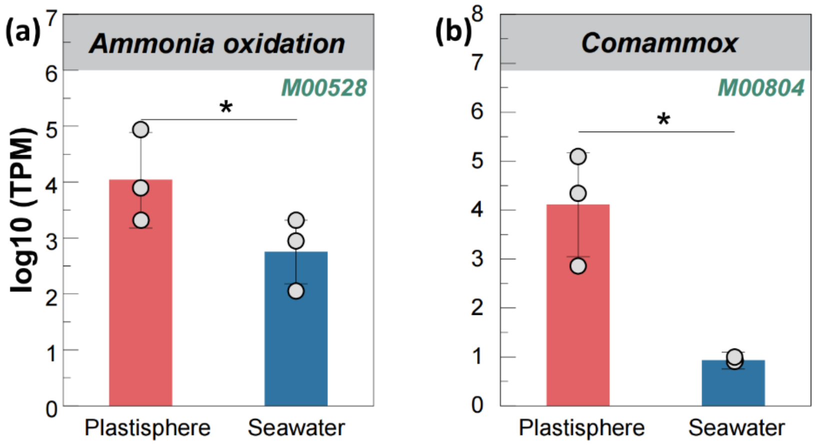

Fig. S16. The expression levels of KEGG modules associated with nitrification process in the plastisphere (P) and surrounding seawater (W) nitrifiers. (a) Ammonia oxidation (M00528, n=3, biological replicates); **(b)** Comammox process (M00804, n=3, biological replicates). No module specifically related to nitrite oxidation was identified in the KEGG database. Data are presented as mean value ± standard deviation. Different letters indicate the significant differences (one-way ANOVA, *P* =0.037 for (a) and <0.001 for (b)).

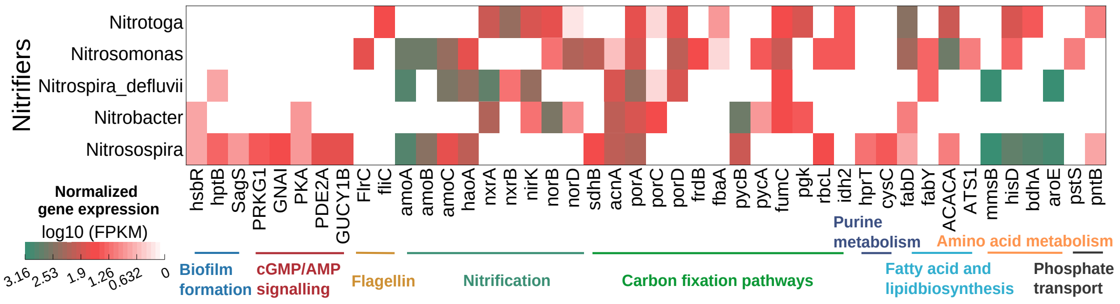

Fig. S17. Gene expression of intracellular metabolisms and nitrification genes of these five nitrifying MAGs. The metabolisms are involved in biofilm formation, cGMP/AMP signalling, flagellin, nitrification, carbon fixation, purine metabolism, fatty acid and lipid synthesis, amino acid metabolism. The normalized gene expression level is based on FPKM.

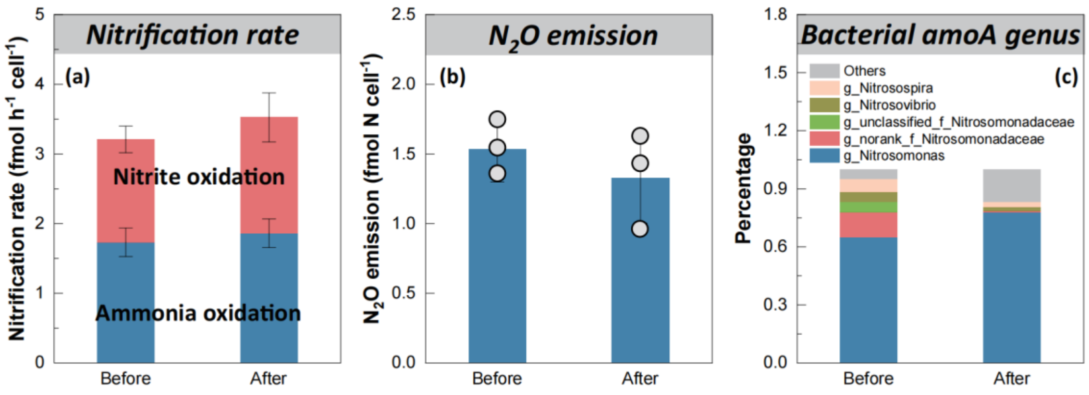

Fig. S18. Nitrification rate, N_2O emission and bacterial *amoA*-based nitrifiers before and after the 28-d *in-situ* incubation. (a) Ammonia oxidation rate (n=3, biological replicates) and nitrite oxidation rate (n=3, biological replicates). **(b)** N_2O emission after 36-h incubation (n=3, biological replicates). **(c)** Bacterial *amoA*-type nitrifier communities at the genus level. Data are presented as mean value ± standard deviation. Different letters indicate the significant differences (one-way ANOVA, $P > 0.05$).

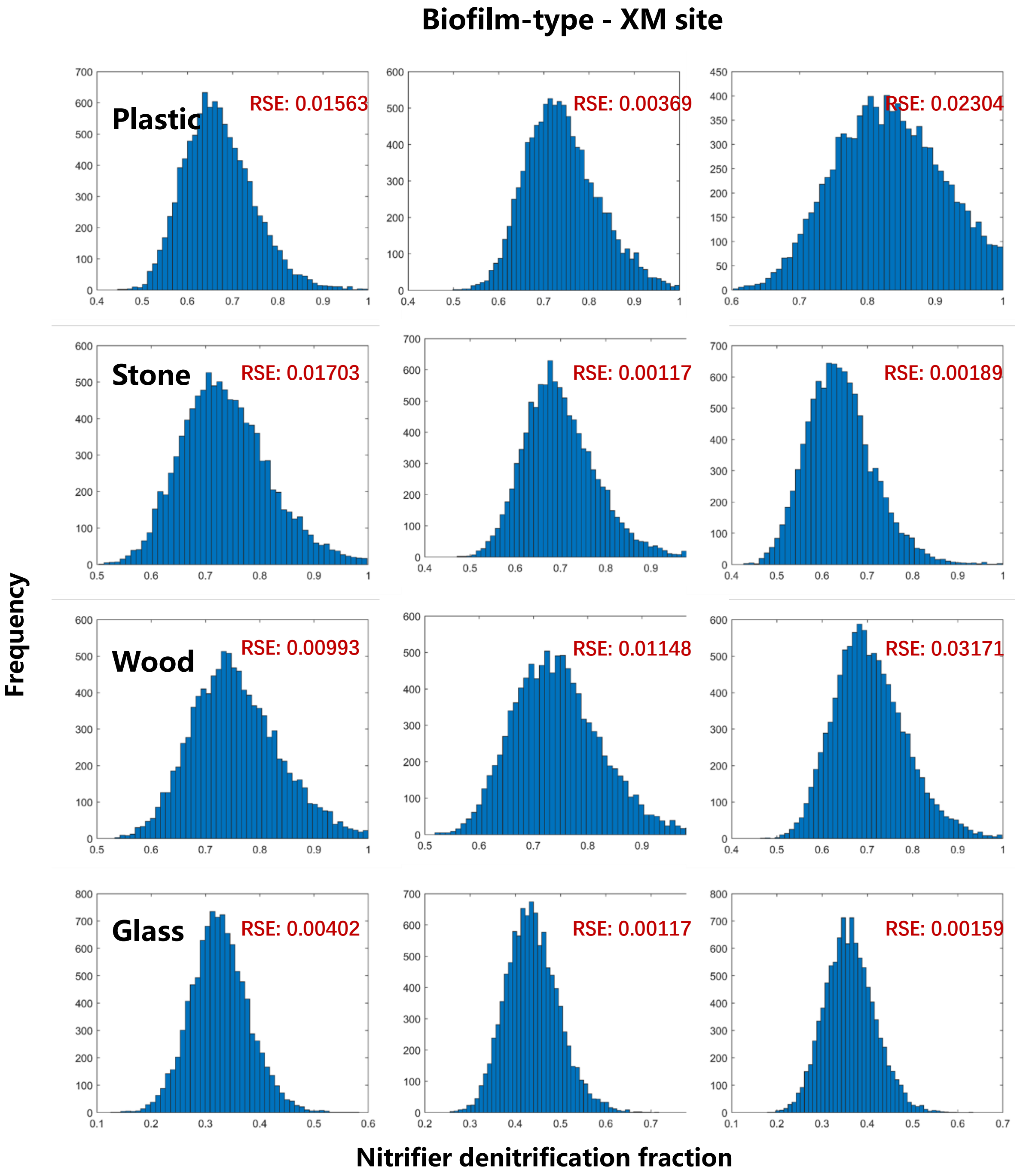

Fig. S19. Monte Carlo stimulation with 10000 times random sampling to assess the calculation errors of N_2O emission pathways in the Experiment 2 (biofilm type). X axis is the proportion of nitrifier denitrification (F_N), and NH_2OH oxidation fraction (F_A) equals to 1-F_N.

To be continued

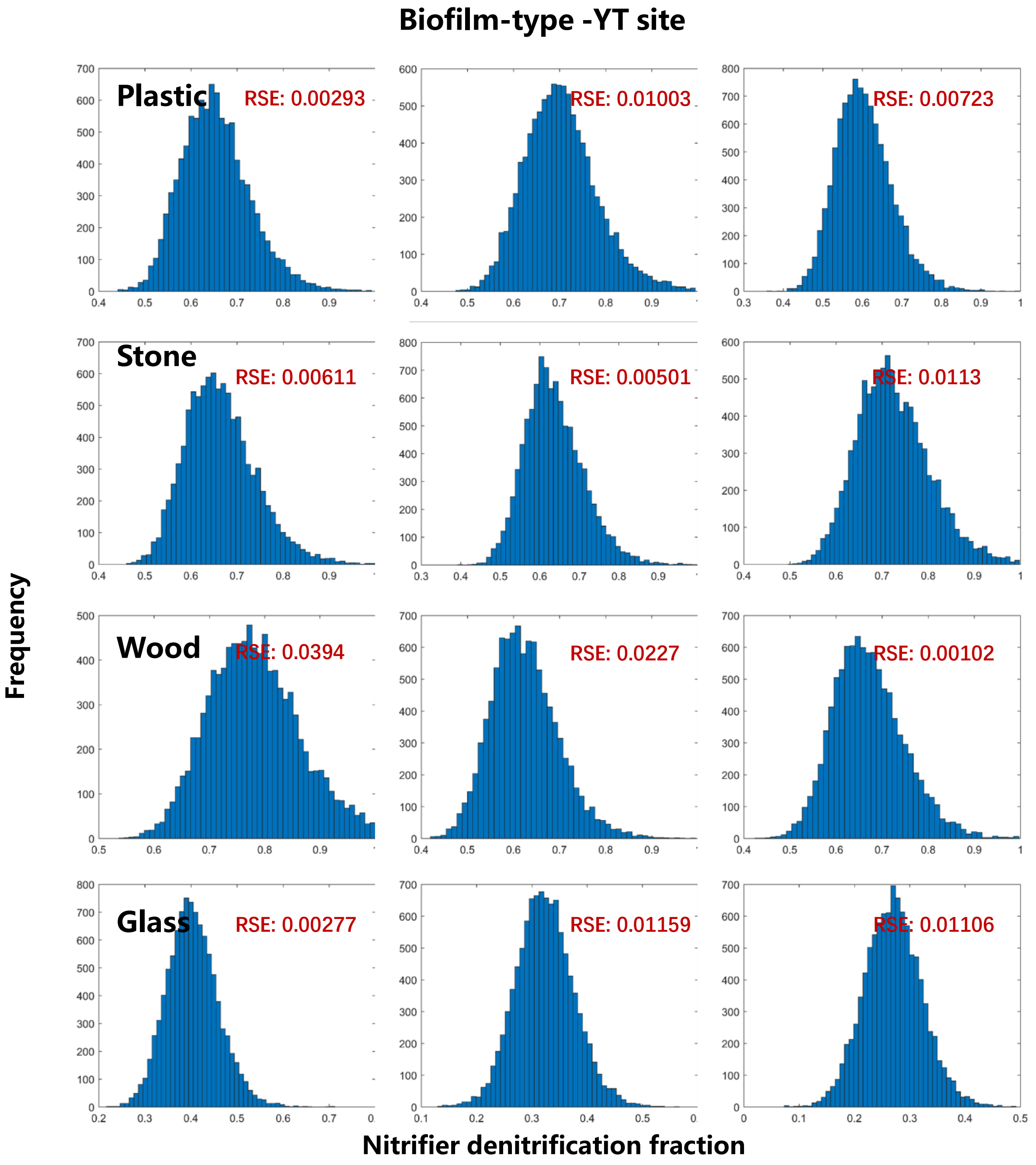

Fig. S19. Monte Carlo stimulation with 10000 times random sampling to assess the calculation errors of N_2O emission pathways in the Experiment 2 (biofilm type). X axis is the proportion of nitrifier denitrification (F_N), and NH_2OH oxidation fraction (F_A) equals to 1-F_N.

To be continued

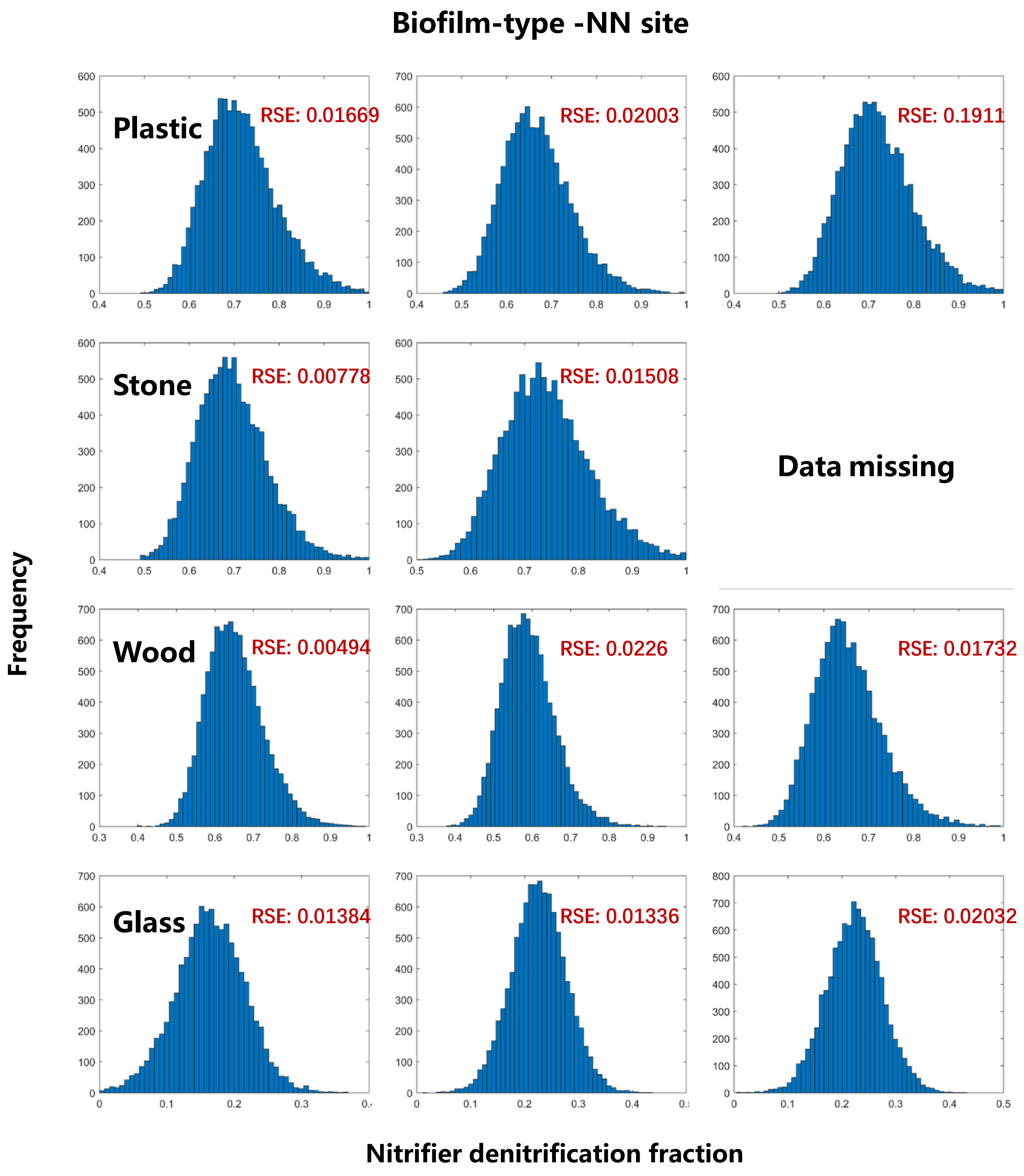

Fig. S19. Monte Carlo stimulation with 10000 times random sampling to assess the calculation errors of N_2O emission pathways in the Experiment 2 (biofilm type). X axis is the proportion of nitrifier denitrification (F_N), and NH_2OH oxidation fraction (F_A) equals to 1-F_N.

To be continued

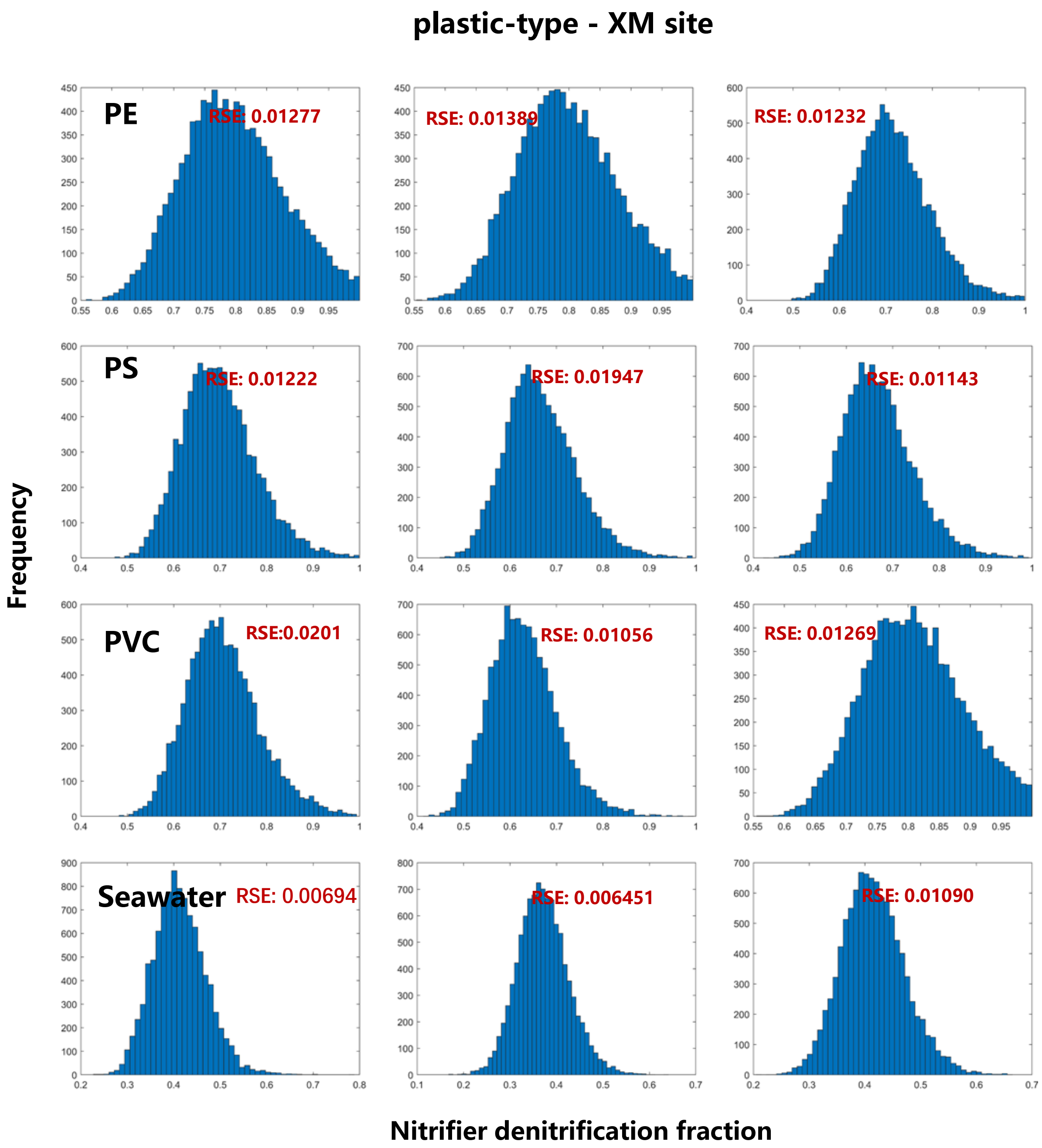

Fig. S19. Monte Carlo stimulation with 10000 times random sampling to assess the calculation errors of N_2O emission pathways in the Experiment 3 (plastic type). X axis is the proportion of nitrifier denitrification (F_N), and NH_2OH oxidation fraction (F_A) equals to 1-F_N.

Supplementary References

1. Sutka RL, Ostrom NE, Ostrom PH, Breznak JA, Gandhi H, Pitt AJ, et al. Distinguishing nitrous oxide production from nitrification and denitrification on the basis of isotopomer abundances. *Appl Environ Microb*. 2006;**72**(1):638-44.

2. Yu L, Harris E, Lewicka-Szczebak D, Barthel M, Blomberg MRA, Harris SJ, et al. What can we learn from N_2O isotope data? – Analytics, processes and modelling. *Rap Commun Mass Spectrom*. 2020;**34**(20):e8858.

3. Frame CH, Casciotti KL. Biogeochemical controls and isotopic signatures of nitrous oxide production by a marine ammonia-oxidizing bacterium. *Biogeosciences*. 2010;**7**(9):2695-709.

4. Wang C, Tang S, He X, Ji G. The abundance and community structure of active ammonia-oxidizing archaea and ammonia-oxidizing bacteria shape their activities and contributions in coastal wetlands. *Water Res*. 2020;**171**.

5. Hou L, Xie XB, Wan XH, Kao SJ, Jiao NZ, Zhang Y. Niche differentiation of ammonia and nitrite oxidizers along a salinity gradient from the Pearl River estuary to the South China Sea. *Biogeosciences*. 2018;**15**(16):5169-87.

6. Maixner F, Noguera DR, Anneser B, Stoecker K, Wegl G, Wagner M, et al. Nitrite concentration influences the population structure of Nitrospira-like bacteria. *Environ Microbiol*. 2006;**8**(8):1487-95.

7. Pester M, Maixner F, Berry D, Rattei T, Koch H, Lucker S, et al. NxrB encoding the beta subunit of nitrite oxidoreductase as functional and phylogenetic marker for nitrite-oxidizing Nitrospira. *Environ Microbiol*. 2014;**16**(10):3055-71.

8. Han S, Luo X, Liao H, Nie H, Chen W, Huang Q. Nitrospira are more sensitive than Nitrobacter to land management in acid, fertilized soils of a rapeseed-rice rotation field trial. *Sci Total Environ*. 2017; **599**:135-144.

9. Liu H, Hu H, Huang X, Ge T, Li Y, Zhu Z, et al. Canonical ammonia oxidizers, rather than comammox Nitrospira, dominated autotrophic nitrification during the

mineralization of organic substances in two paddy soils. *Soil Biol Biochem*. 2021;**156**:108192.

10. Su X, Yang X, Li H, Wang H, Wang Y, Xu J, et al. Bacterial communities are more sensitive to ocean acidification than fungal communities in estuarine sediments. *FEMS Microbiol Ecol*. 2021.

11. Hu W, Ran J, Dong L, Du Q, Ji M, Yao S, et al. Aridity-driven shift in biodiversity–soil multifunctionality relationships. *Nat Commun*. 2021;**12**(1):5350.
