# Supplementary material for: Nitrifying niche in estuaries is expanded by the plastisphere
Source: Nat Commun. 2024 Jul 12;15:5866. doi: 10.1038/s41467-024-50200-8 (PMC11245476; doi:10.1038/s41467-024-50200-8)
Supplement: Supplementary file 3 — Description of Additional Supplementary Files [file 41467_2024_50200_MOESM3_ESM.pdf]

Description of Additional Supplementary Files

File Name: Supplementary Data 1
Description: Information and summary of the metagenome-assembled genome (MAGs)..
