# Supplementary material for: Nitrifying niche in estuaries is expanded by the plastisphere
Source: Nat Commun. 2024 Jul 12;15:5866. doi: 10.1038/s41467-024-50200-8 (PMC11245476; doi:10.1038/s41467-024-50200-8)
Supplement: Supplementary file 5 — Reporting Summary [file 41467_2024_50200_MOESM5_ESM.pdf]

Corresponding author(s): Yong-guan Zhu

Last updated by author(s): Jun 22, 2024

Reporting Summary

Nature Portfolio wishes to improve the reproducibility of the work that we publish. This form provides structure for consistency and transparency in reporting. For further information on Nature Portfolio policies, see our Editorial Policies and the Editorial Policy Checklist.

Statistics

For all statistical analyses, confirm that the following items are present in the figure legend, table legend, main text, or Methods section.

n/a	Confirmed	
☐	☒	The exact sample size (*n*) for each experimental group/condition, given as a discrete number and unit of measurement
☐	☒	A statement on whether measurements were taken from distinct samples or whether the same sample was measured repeatedly
☐	☒	The statistical test(s) used AND whether they are one- or two-sided<br>*Only common tests should be described solely by name; describe more complex techniques in the Methods section.*
☒	☐	A description of all covariates tested
☐	☒	A description of any assumptions or corrections, such as tests of normality and adjustment for multiple comparisons
☐	☒	A full description of the statistical parameters including central tendency (e.g. means) or other basic estimates (e.g. regression coefficient) AND variation (e.g. standard deviation) or associated estimates of uncertainty (e.g. confidence intervals)
☐	☒	For null hypothesis testing, the test statistic (e.g. *F*, *t*, *r*) with confidence intervals, effect sizes, degrees of freedom and *P* value noted<br>*Give P values as exact values whenever suitable.*
☐	☒	For Bayesian analysis, information on the choice of priors and Markov chain Monte Carlo settings
☒	☐	For hierarchical and complex designs, identification of the appropriate level for tests and full reporting of outcomes
☒	☐	Estimates of effect sizes (e.g. Cohen's *d*, Pearson's *r*), indicating how they were calculated

Our web collection on statistics for biologists contains articles on many of the points above.

Software and code

Policy information about availability of computer code

Data collection	Data in this study were collected from IRMS (thermal Delta V), GC (Agilent 7890 A), IC (Dionex CS300), R200 digital refractometer (Reichert, USA), Illumina MiSeq PE300 platform (Illumina, San Diego, CA), PacBio Sequel IIe System (Pacific Biosciences, CA, USA) etc. Other deta,ils see the Materials and methods section.
Data analysis	Most of the analyses were carried out using SPSS (version 22.0) and R (version 3.1.2) softwares; Adobe Illustrator (version CS6) and Origin (version 2019) was used for creating figures; genome assembly and binning were conducted with metaWRAP (version 1.2.1) pipeline; the binning was carried out by the CONCOCT (version 0.4.0), MaxBin2 (version 2.2.2) and MetaBAT (version 2.12.1); the mRNA reads were linked to the nitrifying bins and counted in KALLISTO (version 0.46). Additional details are described in the Materials and methods section.<br>The Codes are in the GitHub website - https://github.com/xuangood/estuarine-plastisphere, and the figshare (DOI: https://doi.org/10.6084/m9.figshare.26087410.

For manuscripts utilizing custom algorithms or software that are central to the research but not yet described in published literature, software must be made available to editors and reviewers. We strongly encourage code deposition in a community repository (e.g. GitHub). See the Nature Portfolio guidelines for submitting code & software for further information.

Data

Policy information about availability of data

All manuscripts must include a data availability statement. This statement should provide the following information, where applicable:

- Accession codes, unique identifiers, or web links for publicly available datasets
- A description of any restrictions on data availability
- For clinical datasets or third party data, please ensure that the statement adheres to our policy

Sequencing data generated in this study have been deposited in the NCBI database under accession number SUB12931929 for amplicon sequencing data, SUB12931935 for metagenome data, and SUB12931940 for metatranscriptome data. All other data of this study are available in Supplementary information, supplementary data, GitHub (https://github.com/xuangood/estuarine-plastisphere) or figshare: MAGs (DOI: https://doi.org/10.6084/m9.figshare.26085544), representative sequences (DOI: https://doi.org/10.6084/m9.figshare.26087422), figures with raw data (DOI: https://doi.org/10.6084/m9.figshare.26087419).

Research involving human participants, their data, or biological material

Policy information about studies with human participants or human data. See also policy information about sex, gender (identity/presentation), and sexual orientation and race, ethnicity and racism.

Reporting on sex and gender	Not involved.
Reporting on race, ethnicity, or other socially relevant groupings	Not involved.
Population characteristics	Not involved.
Recruitment	Not involved.
Ethics oversight	Not involved.

Note that full information on the approval of the study protocol must also be provided in the manuscript.

Field-specific reporting

Please select the one below that is the best fit for your research. If you are not sure, read the appropriate sections before making your selection.

☐ Life sciences ☐ Behavioural & social sciences ☒ Ecological, evolutionary & environmental sciences

For a reference copy of the document with all sections, see nature.com/documents/nr-reporting-summary-flat.pdf

Ecological, evolutionary & environmental sciences study design

All studies must disclose on these points even when the disclosure is negative.

Study description	Here we selected three estuarine regions in China (Figure 1a) and conducted a series of in-situ incubations and lab-scale experiments based on biofilm type (plastic, glass, stone and wood) and plastic type-based (polyethylene, polystyrene and polyvinylchloride) to investigate the nitrification potential, and then to compare the core nitrifiers between the plastisphere biofilms (sessile mode) and surrounding seawater (planktonic mode). The experimental workflow of this study is outlined in Supplementary Figure S1. The specialized features of the plastisphere lead us to hypothesize that (1) the estuarine plastisphere represents an overlooked and even unique niche of nitrification with higher nitrifying activity than the surrounding seawater and other biofilms, and (2) it harbors distinctive active nitrifiers and metabolic behaviors from the seawater. To test the hypotheses, we measured nitrification rates (NH3 oxidation and NO2- oxidation), and N2O emission and related pathways (NH2OH oxidation and nitrifier denitrification) using 15N isotope tracing and N2O isotopocules methods. Next, employing 13C-DNA stable isotope probing (DNA-SIP) and sequencing of amplicons and metagenomes, we identified active keystone nitrifier communities. Finally, we revealed metabolic differences of these active nitrifiers between the plastisphere (sessile mode) and seawater (planktonic mode) using metagenome-assembled genomes (MAGs)-centric metatranscriptomic analyses.
Research sample	In this study, we chose plastic debris (PE, PS, and PVC), stone debris, floating woods, glass balls, and seawater as our research samples. These materials are commonly found in estuarine environments and have been frequently utilized in various research studies.
Sampling strategy	For Experiment 1, 2 and 3, three types of plastic debris, stone debris, floating woods, glass balls and in situ seawater were collected (Sample size n =4 for each, biological replicates. Other details see Methods section).For experiment 4, three types of plastic debris and in situ seawater were used (Sample size n =3 for each, biological replicates. Other details see Methods section). Prior to analysis, we tested for the homogeneity of variances (Levene's test) and the normality of residuals. One-way analysis of variance (One-way ANOVA) combined with the Tukey post hoc test was then performed for the significance test.

Data collection	S.X.X., H.X.R., Y.L.Y, Z.Y.Y. Z.G.B., and Z.Y-G. conceived the study and conducted the incubations, analyzed the data and wrote the manuscript. S.X.X. Z.Y.Y. H.X.R. and T.Y.J. analyzed sequencing data with QIIME 2. S.X.X., H.X.R. and Y.L.Y. measured N speciation with IC. Y.X.R., W.T., P.J.L., and Z.J.B. detected isotopes and analyzed the data with IRMS. D.J. and L.R.L. helped to conduct in-situ incubations in YT and NN sampling sites. S.X.X., L.Z.L., and H.F.Y. conducted DNA-SIP analysis with ultracentrifuge. Y.X.R., T.Y.J, M.W., C.X.P. and M.R. edited the manuscript.
Timing and spatial scale	All samples and experiments were collected and conducted from June to August of 2022 and 2023.
Data exclusions	No data exclusion.
Reproducibility	All measurements were carried out in triplicate or quadruplicate. All samples were repeatedly collected from each plastisphere, stone debris, floating woods, glass balls and surrounding seawater. All attempts to repeat our experiments were successful.
Randomization	Samples for each measurements were randomly collected and allocated in this study.
Blinding	As both the experiments and the analyses were carried out by the same group of scientists, blinding was not relevant for this study.

Did the study involve field work? ☒ Yes ☐ No

Field work, collection and transport

Field conditions	The study areas are located in the estuaries and coasts, including (1) Xiamen (XM), Fujian province (118°11'E, 24°57N); (2) Yantai (YT), Shandong province (121°47E, 37°46N); (3) Nanning (NN), Guangxi province (108°27E, 22°84N). XM and NN have a subtropical climate with 21°C and 22°C mean air temperature and 1000 mm and 1300 mm annual rainfall, respectively. YT has a temperate continental climate with 13°C mean air temperature and 524 mm annual rainfall. These estuaries are influenced by anthropogenic activities (such as input of wastewater, nutrients, heavy metals or other pollutants).<br>We sampled the surface seawater of the three sampling sites from June to August, 2022 and 2023. After collection, the samples were kept in a 4°C ice box and transported back to laboratory. Their water chemical characteristics were measured within 24 hours. XM site (average values (n=3)): pH 6.8, 28°C, 6.98 mg L^{-1} of dissolved oxygen, 72.9 mg L^{-1} of total organic carbon, 1.06 mg L^{-1} of NO3-, 0.26 mg L^{-1} NH4+, 0.06 mg L^{-1} of NO2-. YT site: pH 7.3, 24°C, 7.23 mg L^{-1} of dissolved oxygen, 129.7 mg L^{-1} of total organic carbon, 1.37 mg L^{-1} of NO3-, 0.39 mg L^{-1} NH4+, 0.32 mg L^{-1} of NO2-. NN site: pH 7.1, 27°C, 7.06 mg L^{-1} of dissolved oxygen, 78.4 mg L^{-1} of total organic carbon, 0.79 mg L^{-1} of NO3-, 0.17 mg L^{-1} NH4+, 0.09 mg L^{-1} of NO2-.
Location	Xiamen (XM), Fujian province (118°11'E, 24°57N); (2) Yantai (YT), Shandong province (121°47E, 37°46N); (3) Nanning (NN), Guangxi province (108°27E, 22°84N).
Access & import/export	The collections of samples in this study did not involve sensitive or prohibited areas.
Disturbance	No disturbance was caused by this study.

Reporting for specific materials, systems and methods

We require information from authors about some types of materials, experimental systems and methods used in many studies. Here, indicate whether each material, system or method listed is relevant to your study. If you are not sure if a list item applies to your research, read the appropriate section before selecting a response.

Materials & experimental systems

n/a	Involved in the study	
☒	☐	Antibodies
☒	☐	Eukaryotic cell lines
☒	☐	Palaeontology and archaeology
☒	☐	Animals and other organisms
☒	☐	Clinical data
☒	☐	Dual use research of concern
☒	☐	Plants

Methods

n/a	Involved in the study	
☒	☐	ChIP-seq
☒	☐	Flow cytometry
☒	☐	MRI-based neuroimaging
